# The meaning of quality work from the general practitioner's perspective: an interview study

**DOI:** 10.1186/1471-2296-7-60

**Published:** 2006-10-19

**Authors:** Eva Lena Strandberg, Ingvar Ovhed, Anders Håkansson, Margareta Troein

**Affiliations:** 1 Blekinge R&D Unit, Erik Dahlbergsv. 30, SE-374 37 Karlshamn, Sweden; 2 Lund University, Department of Clinical Sciences, Malmö, Family Medicine, Malmö University Hospital, SE-205 02 Malmö, Sweden

## Abstract

**Background:**

The quality of health care and its costs have been a subject of considerable attention and lively discussion. Various methods have been introduced to measure, assess, and improve the quality of health care. Many professionals in health care have criticized quality work and its methods as being unsuitable for health care. The aim of the study was to obtain a deeper understanding of the meaning of quality work from the general practitioner's perspective.

**Methods:**

Fourteen general practitioners, seven women and seven men, were interviewed with the aid of a semi-structured interview guide about their experience of quality work. The interviews were tape-recorded and transcribed verbatim. Data collection and analysis were guided by a phenomenological approach intended to capture the essence of the statements.

**Results:**

Two fundamentally different ways to view quality work emerged from the statements: A pronounced top-down perspective with elements of control, and an intra-profession or bottom-up perspective. From the top-down perspective, quality work was described as something that infringes professional freedom. From the bottom-up perspective the statements described quality work as a self-evident duty and as a professional attitude to the medical vocation, guided by the principles of medical ethics. Follow-up with a bottom-up approach is best done in internal processes, with the profession itself designing structures and methods based on its own needs.

**Conclusions:**

The study indicates that general practitioners view internal follow-up as a professional obligation but external control as an imposition. This opposition entails a difficulty in achieving systematism in follow-up and quality work in health care. If the statutory standards for systematic quality work are to gain a real foothold, they must be packaged in such a way that general practitioners feel that both perspectives can be reconciled.

## Background

In the last few decades the quality of health care and its costs have been a subject of great attention and lively discussion both in Sweden and internationally. Among other things, this has meant changes to Swedish legislation, which now requires systematic follow-up of the quality of health care [1-3]. The legislation lacks explicit instructions on practical implementation. Responsibility for the choice of method rests with the organization. Consequently, different methods and tools have been introduced to measure, assess, and develop quality in health care in purely general terms [4,5]. One area that has been in particular focus in Sweden is the prescribing of drugs [6-10].

The actual process of following up, assessing, and developing quality has been given several names, which are sometimes used synonymously in everyday speech, such as quality assurance (in use since 1980), quality assessment, quality improvement, quality development and the more overall term quality work. In the following text the term quality work is used as a comprehensive conception throughout.

Many professionals in health care have criticized quality work in the general debate because several of the models and tools introduced are unsuitable for activities in health care [4,11-13]. Alternative methods have been developed by the professionals themselves [14-16]. One method that has proved to work for follow-up and modification of clinical procedure is audit according to the Audit Project Odense (APO) model, extensively used in Denmark and to a limited extent in Sweden, Norway and Iceland [17-20]. Similar approaches are to be found in many other countries, both in Europe and worldwide [21-24]. The conception 'Medical Audit' was introduced in 1968 and the definition in the MeSH Database is 'A detailed review and evaluation of selected clinical records by qualified professional personnel for evaluating quality of medical care'. In connection with the authors' involvement in various audit projects, the question of general practitioners' perceptions of quality work arose.

The aim of the study was to obtain a deeper understanding of the meaning of quality work from the general practitioner's perspective.

## Methods

To arrive at an understanding of quality work from the general practitioner's perspective we chose a qualitative approach using semi-structured interviews with open-ended questions, in accordance with Kvale [25]. The interviews were designed against the background of the authors' pre-understanding of the research field. ELS, who conducted all the interviews, works at the Blekinge Research and Development Unit, developing methods and activities in primary care; she also has experience of quality work and process supervision from various audit projects. The other authors are general practitioners and researchers.

### Data collection

We made a strategic selection of informants so that we could expect a wide spread of experiences and perceptions as regards age, gender, and form of organization. We sent written invitations to eight doctors who had taken part in an audit on the prescribing of psychotropic drugs and the six doctors from the same health care district that had no experience of audit projects. All eight audit participants and two of the non-participants agreed to be interviewed. The remaining four non-participants had terminated their employment without stating new addresses. To increase the number of informants without experience of audit, general practitioners were invited from a neighbouring district. Four doctors agreed to be interviewed. A total of 14 persons were interviewed (Table 1).

**Table 1:** Characteristics of the informants

	Experience of audit	No experience of audit
Men	4	3
Women	4	3
Public employees	6	5
Private practice	2	1
Age range	49–60	35–64
Mean age	54	45

The interviews were held at each doctor's office. To begin with we had two pilot interviews but they were not included in the analysis. The interviewer had no professional ties with any of the respondents, nor did the co-authors.

The interviews comprised questions about the statutory requirement for quality work, what it meant for the doctor, and how the doctor felt that it affected the patient and the health care principal. The doctors were also asked whether and if so how they followed up their work and about their experience of auditing.

The informants interpreted the questions themselves and were able to talk about their personal experiences of quality work in as concrete terms as possible. Through supplementary questions the interviewer encouraged them to greater clarity. The interviews lasted between 45 and 90 minutes each. They were tape-recorded and then transcribed verbatim by a secretary. In connection with each interview, supplementary notes were taken about the actual interview situation. These were added to each interview as special memoranda.

#### Ethical considerations

Participation was voluntary. All participants gave their consent to participate by replying to the written invitation. All data were treated confidentially and could not be traced to any named person.

### Data analysis

The collection and analysis of data were guided by a phenomenological approach in order to capture the essence of quality work from the general practitioner's perspective and to bring out its characteristics [25-27].

The interviewer (ELS) listened through all the interviews together with the transcript and amended the text where the secretary had misunderstood or where the recording had not been perfect. Two of the authors (ELS, MT) analysed the transcribed interviews separately by first reading the 14 interviews to gain an idea of the totality. The interviews were then analysed in order to discern significant meaning units in ideas and aspects of quality work, which were marked in the text. These were grouped and then brought together in categories by ELS and MT together.

Each category was named for its main content, after which the categories were grouped according to themes. The authors discussed ambiguities and obscurities in the analysis until consensus was reached, after which the different themes of the interviews were combined in a descriptive statement. During this transformation process the authors tried as far as possible to ignore their own experience (bracketing). Examples of the meaning units, aspects, categories, and themes that were created are presented in Table 2.

**Table 2:** The different steps of the analysis

Examples of			
**Meaning units →**	**Aspects →**	**Categories →**	**Themes**
"That's what you want after all, a bit of guidance so that there's some equality"	Care on equal terms	Reasons	Top-down
"Otherwise you risk being reported for what you do or don't do ... and even if you don't think about it all the time, it's something you know about ... and that means that, besides having a general desire to do good, so to speak, you naturally have it as a reason to try to stay updated and do the right things"	Good care		
"We do it mostly because you've got to have something, it's what you're supposed to have according to the National Board of Health and Welfare"	External compulsion		
	
"It has meant that I have been on quality courses and I've sat on the quality group at the County Council, where they worked with Quality, Development, Leadership, which I was dubious about. Far too big and too ... it didn't suit our work in primary care"	Externally imposed control system (package solutions)	Methods	
"Well, if we are to return to our own quality assurance system, here are 21 points, this here, our very own system that we sometimes take out to see if we fulfil ... and then we go through it and reflect, have we done this and that, does this work, have we written up, and things like that"	Locally developed control system		

"You try all the time to keep updated when it comes to local, national, and international care programmes and the like, we try to work with the medical quality"	Internal demands	Reasons	Bottom-up
"One effect that is maybe not so noticeable, but I myself feel more secure in my own role, and I believe that I pass that on to the patients too: I know this is the best thing for you"	Good and equal care		
	
"You do that all the time ... with the individual patient. Find out, follow up, because I meet each patient all the time"	Own follow-up	Methods	
"... we have at least an hour allocated each week when all the colleagues meet and you have a chance to present cases and get comments"	Collegial comparison		

The other two authors (IO and AH) read four randomly selected interviews and confirmed that they contained data supporting the main findings.

## Results

**Figure 1:**
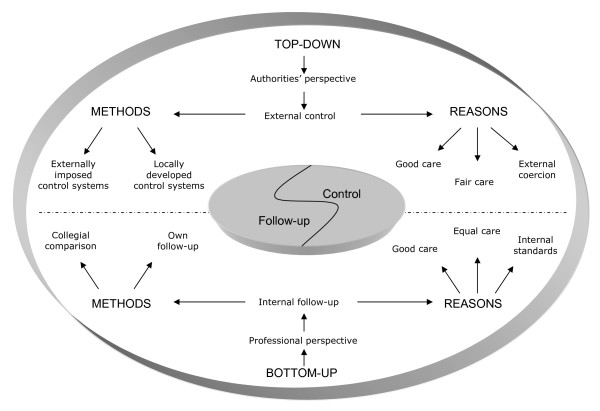
General practitioner's quality work.

Our informants point out two fundamentally different ways of viewing work with quality: a pronounced top-down perspective with elements of control, and an opposite intra-profession or bottom-up perspective (Figure 1). Among our informants there were people both with and without experience of audits. This fact did not affect the actual main finding, the top-down and bottom-up perspectives. All respondents made statements belonging to both perspectives. Top-down seemed alien while bottom-up was natural. All the informants also had much more to say about the bottom-up perspective than about the top-down one. Supporting quotations for each perspective are presented in Tables 3 and 4.

**Table 3:** Top-down – a coercive and demanding imposition

**Reasons for and against**
"It *(quality work) *is mostly something we do because you've got to have something, since we *(the organization) *are supposed to have something according to the National Board of Health and Welfare."
"The power of the patients has got bigger and bigger, they feel that they have that support from ... well, I don't know where they have the support from, whether it's from the regulations of the National Board of Health and Welfare or what it is, but they have it ... I suppose it has changed a bit here. The patient's position has really been strengthened a lot."
"I suppose that's what you want *(as a doctor) *after all, a bit of guidance so that there's some equality."
"Just like so many other things, this is very much imposed top-down, but that doesn't change very much in practice."
"What little you can control you are very concerned about ... if something comes from outside that you suspect is supposed to control you, then there's a great risk that you'll show your claws."
"The resistance concerns time above all. You feel that it takes time away from time that you don't have."

**The methods**

"I think it would be more appropriate to call it a control system."
"Far too big and didn't suit our work, in primary care."
"This here *(quality assurance system)*, our very own system that we sometimes take out to see if we fulfil ... and then we go through it and reflect, have we done this and that, does this work, have we written up, and things like that."
"We have some systematic work locally at the health centre too, you could say, for we have a quality council and we work with things here at the health centre that we perceive as important."

**Table 4:** Bottom-up – a natural and self-evident task

**The reasons**
"Besides having a general desire to do good, so to speak, you naturally have it as a reason to try to stay updated and do the right things."

**The methods**

*Prescription statistics* "It's rather useful, for sometimes you believe something but then you can have hard facts about what you prescribe."
*Computer records* "What I would like to use is this box. But we've been feeding in things for six years now, but we never get anything out."
*Personal follow-up* "because I meet every patient all the time, I meet each patient continuously year after year, so I have a continuous assessment of how the patient, how things have gone as a result of the way I handled this, which gives a different longitudinal follow-up from what these spot tests do, you know."
*Collegial comparison* "I always think it's good that you have somebody and can discuss patients with and get new ideas and hear how other people think, what you would have done in this situation, if you would have done what I did, I think that helps me to develop, so that I don't get stuck in different treatment methods. There's a colleague in primary care, an older colleague who must have worked 25 years longer than I have, and I've heard that he's very good and knows his stuff, you know, in every way very competent."
*Audit* "Say that you have some kind of idea about what you normally do in certain situations and perhaps that you don't always remember what compromises you make when you do it differently."
"This thing of comparing yourself with others is interesting to discuss, since we work so much on our own, you know, you don't really know so much about what your colleagues do, you guess and I think that's an important part of it, that we compare ourselves, that we look at how the others do it and how I do it."
*Colleague groups* "We have FQ groups and there I think we have a certain amount of quality assurance, for we sit and discuss how we treat our patients."

### Top-down

From the general practitioners' descriptions, the top-down perspective on quality emerged as a factor that infringed professional freedom. In this perspective, quality work is characterized by coercive impositions, moreover often in forms that are inappropriate for primary care. They are perceived to be about external and time-consuming processes, which generate resistance instead of involvement and participation. The methods introduced often feel like package solutions which are both alien and impractical for the activities. They are not infrequently borrowed from some other sphere than primary care, sometimes from business where the requirement of cost-efficiency is particularly obvious, and sometimes from other fields of health care. For this reason, local control systems have been devised in some places. The imposed systems and methods measure the wrong things, give the greatest guilt feelings and the lowest participation. Several informants believed that these activities do not affect how the work is done; they are perceived mostly as parts of control systems and not as systems for development. A general practitioner with a strictly steered working day reacts negatively against anything that is perceived as control. Quality work in the top-down perspective is felt to steal time from the important work with the patients. Despite personal resistance, there nevertheless seems to be a certain degree of acceptance for the coercive top-down quality work. The reasons stated in this case are that it ensures that society's requirement of good and fair care is satisfied.

### Bottom-up

The bottom-up perspective on quality work emerged from the statements as a self-evident duty and as a professional attitude to medicine, guided by the ethical principles of respect for the individual, of doing good and not doing harm. It is natural to follow up one's own clinical actions in accordance with these principles. This kind of follow-up is best done in internal processes – individually or in groups – and by designing both structures and methods according to the needs that doctors themselves feel that they have. The degree of "ownership" is thus high.

#### Individual self-follow-up

The doctors spoke of the importance of follow-up of treatment when one feels uncertain or not wholly certain. The follow-up of his/her own actions that the doctor does then takes place at different levels and with differing focus, at individual patient level and at patient group level. At group level it is a matter of obtaining a picture of how one acts oneself, and at individual level the aim is to ascertain how the doctor's decisions affect the individual patient. Actual patient contact, both by telephone and in face-to-face encounters, is therefore an excellent follow-up instrument. Writing the patient records oneself and signing them is also a way to follow up the individual patient. Retrospectively going through patient records and keeping personal statistics on one's clinical behaviour or keeping notes about things that are of particular interest and storing them in the desk drawer are useful methods at patient group level. Expectations of computerized records were high, but they have not been fulfilled, partly because it is difficult to extract data, partly because of inadequate training. The computerized records were quite simply said not to have been designed for extracting useful information in an easy way.

#### Follow-up through collegial comparison

Collegial comparison in the form of comments and discussions with colleagues about individual patients was considered particularly valuable, perhaps the most valuable form of professional development. A special form of collegial discussion is mentorship, with an older, more experienced colleague. Medical audit and other forms of statistics are excellent for studying one's own acts in relation to those of colleagues. The focus in these contexts is not on the patient but on the doctor himself. Discussions between doctors working together at a health centre are a third form of collegial comparison. A special variant of collegial discussion is the FQ (further education and quality) group.

Several informants felt that audit according to the Odense model reveals defects and myths in one's own behaviour. The incentive for taking part in such audit projects is precisely the actual comparison with colleagues, above all in the vicinity, with colleagues both in primary care and at the hospital. This gives a sense of participation in a development process. Among those who lacked experience of this type of audit, however, there was a fear that the method could be used as a control instrument and thus belong to the top-down perspective.

## Discussion

The picture that emerged from our informants' statements was unanimous. The doctors made a clear distinction between top-down and bottom-up as partly incompatible perspectives on quality work.

### Methodological considerations

The aim of the study was to seek a deeper understanding of quality work from the general practitioner's perspective. We therefore had a broad recruitment of informants as regards experience, age, gender, form of employment, and experience of audit. The doctors who took part in our study were general practitioners with long experience of Swedish primary care. All were or had previously been public employees, although at the time of the interviews a few were privately practising and had contracts with the health care principal.

We chose a phenomenological approach in order to try to understand general practitioners' ideas about quality work. It might be objected that we guided the informants towards the use of audit, partly through the written invitation to the interview, partly through our semi-structured conversation guide. The audit served as a starting point for planning the study but was not a special interest. The aim, however, was that the informants should be able to speak completely freely. In the invitation we referred to the audit project about the prescribing of psychotropic drugs in which the majority had been invited to participate, but this did not affect the main findings; two opposite perspectives on quality work and the need for both the top-down and bottom-up perspectives for a systematic quality work.

A uniform picture emerged from the statements. The findings, however, cannot be generalized, but this is never the intention in phenomenological studies, the main aim of which is to contribute to increased understanding. It is nevertheless reasonable to assume that our findings can be transferred to similar contexts and that general practitioners with similar working conditions have opinions like these.

### Top-down – a demanding imposition

What our informants call quality work was regarded as an imposition, with coercion and control. The legislator demands systematism, but does not indicate any methods [1-3]. An alternative would be to follow the example from the UK, where quality indicators and quality targets are set in order to be able to quantify the health gain to a practice population [28]. The criticism expressed by our informants was that the methods introduced felt like package solutions, rarely suitable for primary care and sometimes unsuitable for health care as a whole. In a dissertation about quality assurance in health care [4] the author stresses the importance of not uncritically adopting concepts for quality assurance and quality development created in one context and transferred to a different context with wholly or partly different conditions [4]. Here quality assurance is compared to Pandora's box, as the good things in the box changed to evil if it was opened where it did not belong. A model originating in a different organizational field than medicine and care proceeds from a different logic and other values. Models with their origin in business are mainly visible at administrative levels and do not seem to gain a foothold in practice. Work with quality assurance thus does not become a living instrument but is instead regarded as an administrative imposition with no real meaning [11,12]. As a consequence, people at some places in primary care have devised their own systems which better illuminate what general practitioners perceive as important.

### Bottom-up – a self-evident task needing various tools

Follow-up of clinical procedure in general and of prescribing habits in particular was considered a natural part of the professional development and a means to give the patient optimum care [15-17]. Whereas the statutory requirements of systematic quality work were considered as a time-consuming imposition, the professional need to work with evidence-based medicine and to follow up one's own actions was regarded as the natural way to promote development. The profession has also devised its own methods and strategies for this task [14,29,30].

Our informants spoke with great involvement about different ways of following up their prescribing habits. Audit according to the Odense method is based entirely on voluntariness and is thus not a part of the control apparatus [17,18]. Audit as a method for following up one's own habits was therefore considered suitable in primary care; it was felt to be an instrument that supported the doctor's professional autonomy, as also corroborated by Danish and Swedish studies [10,20,31,32]. Studies in the UK, where audit is often associated with a top-down approach, have shown that discussions about audit projects and a positive attitude in the group of colleagues support both participation in and completion of audit projects [33-35]. Although Danish and Swedish studies have shown that audit according to the APO method led to changed behaviour in the participating doctors, other studies have shown that the effect of an audit with feedback is usually rather small; it is greatest in cases where compliance with recommendations is low [36,37]. Veninga et al showed in a study published in 2001 that indicators based on self-report instruments seem to overestimate guideline adherence [38]. According to Grimshaw et al. there is an imperfect evidence base to support decisions about which guideline dissemination and implementation strategies are likely to be efficient under different circumstances. Decision makers must carefully consider how to best use resources for quality improvement activities [39]. It is then interesting to note that our informants stressed collegial discussion in the audit process and in other contexts as by far the most important instrument when it comes to a professional attitude to the patient and everyday clinical work, which relates well to educational theories about principles in adult learning; effective change in health care is achieved better by focussing on concrete problems in practice; professionals are more motivated to change by internal motivation than by external pressure [40].

Computerized medical records have not yet lived up to the expectations that preceded their introduction. Although several studies have shown areas where the use of computerized records works well, they have also demonstrated a need to develop and improve existing systems [41-44]. Our informants are still waiting for computerized records to give back information about prescribing habits, referral procedures, and other types of desirable statistics. The criticism concerned the poor design of a system that does not allow easy extraction of useful information, that it takes too long to produce the desired statistics, and that no training has been given in the use of the system. It is assumed that people will learn this by themselves, which reduces the time left for work with patients. If quality work with the aid of computer support is to become an integral part of the general practitioners' everyday activities, support systems must be designed so that they really do provide support and the training issue must be taken seriously [41]. More knowledge is probably also needed about how human behaviour is affected by the introduction of new systems if computerized medical records are to have their full impact [4,11].

A recurrent theme in our informants' statements was the significance of colleagues for development and learning. Collegial follow-up can take place in many ways. Our informants mentioned in particular various audit projects, inside or outside the FQ group, as good examples of a form of learning in which discussion with colleagues is the main point [29]. The idea of the FQ group is to constitute a forum for collegial discussion. The initiative for establishing such groups in Sweden was taken by the Swedish Association of General Practice (SFAM) around 1990 [45].

### Top-down versus bottom-up – two sides of the same coin?

The question is how incompatible the perspectives actually are. What quality work in health care is ultimately about is the continuous measurement, assessment, analysis, and if necessary improvement of care as a whole, in which diagnosis and treatment are self-evident parts [46]. It is also in the provision of good care that the point of contact exists between statutory requirements and implicit professional duty. Systematism is needed both generally in the organization and specifically for individuals in the organization, in our case the doctors, to avoid *ad hoc *implementation of quality work, which is once again stressed in the National Board of Health and Welfare's newly compiled instructions on management systems for quality and patient security in health care [3]. Quality work mainly consists of two parts: assessment and improvement of quality. Each part has its methods. The medical profession has for a very long time taken the responsibility – often including personal responsibility as well – for ensuring that the care provided is of good quality. This responsibility also includes responsibility for research and development, which are typical characteristics of a profession and its autonomy. Having control over working hours and being able to retain clinical self-determination are other factors that, if missing, have a negative influence on job satisfaction [47].

All quality work proceeds from the current situation, regardless of which level or which individuals in the health care organization are concerned. However, the approach selected and hence the methods used vary. Most such approaches have not been scientifically tested or proved to work; their use is based more on good faith [29]. Other research shows that, in order to achieve real change, a single method is not enough; it takes strategies with different combinations of methods and interventions [22,23,36,38,40,48,50]. Reaching quality targets such as set in the new GP contract in the UK can result in significant health gains, which points towards a need for Top-down approaches [28].

## Conclusions

This study contributes to an increased understanding of the complexity and variety of quality work for the doctor. The study indicates that general practitioners view it as a professional obligation to follow up their own work, but they regard quality work as an imposition and a control function. This antithesis entails a difficulty in implementing systematic follow-up and quality work. The study also indicates the need to adjust methods so that the degree of ownership is as high as possible. If the statutory requirements for systematic quality work are to gain a real foothold, it must be packaged so that general practitioners feel that both perspectives are compatible.

## Competing interests

The authors declare that they have no competing interests.

## Authors' contributions

ELS was involved in study design, conducted and analysed interviews, wrote the paper and will serve as guarantor for the integrity of the data. MT was involved in study design, analysed interviews and contributed to editing the paper. AH was involved in study design and contributed to editing the paper. IO contributed to editing the paper. All authors read and approved the final manuscript.

## Pre-publication history

The pre-publication history for this paper can be accessed here:


